# Enhancement of ^211^At Uptake via the Sodium Iodide Symporter by the Addition of Ascorbic Acid in Targeted α-Therapy of Thyroid Cancer

**DOI:** 10.2967/jnumed.118.222638

**Published:** 2019-09

**Authors:** Tadashi Watabe, Kazuko Kaneda-Nakashima, Yuwei Liu, Yoshifumi Shirakami, Kazuhiro Ooe, Atsushi Toyoshima, Eku Shimosegawa, Mitsuhiro Fukuda, Atsushi Shinohara, Jun Hatazawa

**Affiliations:** 1 Department of Nuclear Medicine and Tracer Kinetics, Osaka University Graduate School of Medicine, Osaka, Japan; 2 Core for Medicine and Science Collaborative Research and Education, Project Research Center for Fundamental Sciences, Osaka University Graduate School of Science, Osaka, Japan; 3 Institute for Radiation Sciences, Osaka University, Osaka, Japan; 4 Department of Molecular Imaging in Medicine, Osaka University Graduate School of Medicine, Osaka, Japan; 5 Research Center for Nuclear Physics, Osaka University, Osaka, Japan; and; 6 Department of Chemistry, Graduate School of Science, Osaka University, Osaka, Japan

**Keywords:** astatine, thyroid cancer, sodium iodide symporter, alpha therapy, ascorbic acid

## Abstract

^211^At is an α-emitter that has similar chemical properties to iodine and is used in targeted α-therapy. In the present study, we added ascorbic acid (AA) to ^211^At solution to increase the radiochemical purity of astatide and evaluated its efficacy against differentiated thyroid cancer, which is characterized by the expression of sodium/iodide symporter (NIS). **Methods:** Crude ^211^At solution (AA(−)) and ^211^At solution treated with AA (AA(+)) were prepared. Uptake by the thyroid was compared between the 2 solutions in normal male Wistar rats (*n* = 6). Cellular uptake in K1-NIS cells was analyzed under the AA(+) and AA(−) conditions. AA(+) was injected at 3 doses into K1-NIS xenograft mice: 1 MBq (*n* = 6), 0.4 MBq (*n* = 6), and 0.1 MBq (*n* = 6), and vehicle was injected into control mice (*n* = 6). The treatment effects were compared among the 4 groups. **Results:** Uptake by the thyroid was significantly enhanced in rats injected with the AA(+) as compared with those injected with AA(−). Cellular uptake analysis showed significantly increased uptake of ^211^At by the K1-NIS cells under the AA(+) condition as compared with the AA(−) condition. In the mouse xenograft model, the K1-NIS tumors showed significant accumulation of ^211^At at 3 and 24 h after administration (22.5 ± 10.4 and 12.9 ± 6.8 percentage injected dose, respectively). Tumor growth was immediately inhibited in a dose-dependent manner after administration of ^211^At. In the survival analysis, the ^211^At groups (0.1, 0.4, and 1 MBq) showed significantly better survival than the control group. **Conclusion:** Uptake of ^211^At was enhanced in differentiated thyroid cancer cells as well as the normal thyroid using ^211^At solution treated with AA. The method also showed dose-dependent efficacy against the K1-NIS xenografts, suggesting its potential applicability to targeted α-therapy.

Radioactive iodine has long been used clinically for patients with differentiated thyroid cancer (1–3). ^131^I is used for the ablation of thyroid remnants or treatment of metastatic thyroid cancer (1). However, some patients with multiple metastases are refractory to repetitive ^131^I treatment, despite the targeted regions showing sufficient iodine uptake (4,5). According to the criteria described in the 2015 American Thyroid Association guidelines, radioactive iodine–refractory cancer includes metastatic disease that progresses despite showing substantial uptake of radioactive iodine (6). In such patients, β-particle therapy using ^131^I is inadequate and another strategy is needed using a more effective radionuclide targeting the sodium/iodide symporter (NIS).

Astatine (^211^At) is receiving increasing attention as an α-emitter for targeted radionuclide therapy (7–9). ^211^At is a halogen element with similar chemical properties to iodine (10). α-particles emitted from ^211^At with a branching ratio of 41.8% (5.98 MeV) have higher linear energy transfer than β-particles from ^131^I (0.97 MeV) and exert a better therapeutic effect by inducing DNA double-strand breaks and free radical formation (11). Petrich et al. reported that ^211^At accumulated in the normal thyroid and in tumor xenografts of a genetically modified NIS-expressing papillary thyroid cancer cell line (12). They also showed that ^211^At was effective against thyroid cancer xenografts (12). Meanwhile, the oxidative states of ^211^At solutions may vary and the chemical form and dynamics in the body of ^211^At have not yet been clearly elucidated, because there is no stable isotope of ^211^At (13,14). It is presumed that a plenitude of astatine species, such as At^+^, AtO^−^, At(OH)_2_^−^, AtO_2_^−^, AtO(OH)_2_^−^, and AtO^+^, as well as At^−^, are present in basic or acidic aqueous solutions under an oxidative or reductive condition. In many radiopharmaceutical solutions, ascorbic acid (AA) is frequently added as a stabilizing agent since it is vitamin C and can safely be administered in humans. There is a possibility of stabilizing the oxidative states of ^211^At in its solutions by adding an appropriate reducing agent. In the present study, we prepared ^211^At solutions focusing on the radiochemical purity of astatide (or astatide ion) and evaluated its distribution in normal thyroid tissue via transport through NIS, as well as its efficacy against differentiated thyroid cancer in a tumor xenograft model.

## MATERIALS AND METHODS

### Preparation of ^211^At Solutions

^211^At was produced by the ^209^Bi(α,2n)^211^At reaction, followed by separation and purification by a dry distillation method. ^211^At was dissolved in 100 μL of distilled water. The ^211^At-crude solution at a final concentration of 10 MBq/mL was mixed with AA as a reducing agent at a final concentration of 1.2 w/v% and sodium bicarbonate as a pH adjuster at a final concentration of 2.1 w/v% at pH 8.0 and allowed to stand for 1 h at ambient temperature. Separately, the ^211^At-crude solution was also mixed with the other reducing agents, namely cysteine, glutathione, sodium sulfite, or ferrous sulfate, at a final concentration of 1 w/v% under the same conditions. The resultant mixtures of ^211^At solutions were analyzed by thin-layer chromatography (TLC) using Typhoon 7000 (GE Healthcare). The reagents were purchased from Nacalai Tesque.

### In Vitro Cellular Uptake and Survival Analysis

K1 cells (human papillary thyroid carcinoma) were provided by the European Collection of Authenticated Cell Cultures. K1 cells were maintained in culture medium, D-MEM:Ham F12:MCDB 105 (2:1:1) supplemented with 2 mM glutamine and 10% heat-inactivated fetal bovine serum. K1-NIS cells were obtained by transfection using the human SLC5A5 (NIS) gene clone (OriGene). K1 cells and K1-NIS cells were seeded onto a 24-well plate (1 × 10^5^/well) and cultured for 2 d. After treatment of ^211^At, the cells were washed twice with phosphate-buffered saline(−) and the radioactivity was measured using a 2480 Wizard^2^ γ-counter (Perkin Elmer).

K1-NIS cells were seeded onto a 96-well plate (2 × 10^4^/well) and cultured for 2 d. ^211^At and ^131^I were serially diluted with the culture medium (100 μL/well). After 48 h of incubation, we measured cell viability using Cell Counting Kit 8 (Dojindo).

### Preparation of Animals

Normal male Wistar rats and male ICR and SCID mice were purchased from Japan SLC Inc. and Charles River Japan, Inc., respectively. Animals were housed under a 12-h light/12-h dark cycle and given free access to food and water. The Wistar rats were fed with a low-iodine diet during the 2 wk before the experiment to make the thyroid condition uniform, in accordance with the method used in a previous study (15). Tumor xenograft models were established by subcutaneous injection of K1-NIS cells (1–2 × 10^7^ cells) suspended in 0.2 mL of culture medium and Matrigel (1:1; BD Biosciences) into the SCID mice.

All animal experiments complied with the guidelines of the Institute of Experimental Animal Sciences. The protocol was approved by the Animal Care and Use Committee of the Osaka University Graduate School of Medicine. The criteria for euthanasia were as follows: when the animals had intolerable suffering, when a significant decrease in activity or a marked decrease in food and water intake was observed, or when the end of the observation period was reached (up to 84 d). Euthanasia was through deep anesthesia by isoflurane inhalation.

### Administration of ^211^At Solutions and Imaging Analysis

Two groups of normal Wistar rats (*n* = 6; 12 wk old; body weight, 295.2 ± 16.2 g) were anesthetized with 2% isoflurane and injected with the ^211^At solutions (AA(−), 3.58 ± 0.65 MBq, or AA(+), 2.72 ± 0.12 MBq) through the tail vein. Normal ICR mice (*n* = 11; 10 wk old; body weight, 37.9 ± 1.6 g) were used for the evaluation of toxicity at 3, 7, and 15 d after administration of AA(+) (1.00 ± 0.16 MBq).

K1-NIS tumor xenograft mice (*n* = 24; 10 wk old; body weight, 21.4 ± 1.92 g) were investigated 37 d, on average, after implantation, when the tumor size reached approximately 10 mm in diameter. Under 2% isoflurane anesthesia, K1-NIS mice were injected with AA(+) through the tail vein. Mice were divided into 4 groups according to the injected dose (1 MBq [*n* = 6, 0.99 ± 0.09 MBq], 0.4 MBq [*n* = 6, 0.39 ± 0.13 MBq], 0.1 MBq [*n* = 6, 0.11 ± 0.07 MBq], and control [*n* = 6]). In the control group, vehicle solution and AA were administered.

Planar and SPECT images were acquired with a γ-camera system (E-cam; Siemens) with a low-energy all-purpose collimator (16). The energy window was set at 79 keV ± 20% targeting the x-rays emitted from the daughter nuclide of ^211^Po (17). The radioactivity in the major organs was measured with a γ-counter after euthanasia and dissection at 24 h. Regions of interest were placed using AMIDE software (version 1.0.4). Radioactivity levels in the major organs were measured with a γ-counter after euthanasia and dissection at 24 h. Uptake was normalized by the injected dose (MBq) and body weight (g). The equivalent dose (Gy) in the dosimetry of ^211^At was estimated according to a previous report (18). Tumoral uptake was estimated from the planar images at 3 and 24 h after injection, and the area under the curve after 24 h was assumed to decrease with physical decay.

### Histologic Analysis

After the animals were sacrificed by euthanasia, the tumor, thyroid, and stomach were resected. The specimens were fixed overnight with 4% paraformaldehyde and cryoprotected in 30% sucrose in phosphate-buffered saline. Frozen sections of the samples were then incubated with NIS-antibody (Anti-SLC5A5, Rabbit-Poly; Atlas Antibodies). Immunohistochemistry was performed using the EnVision+ system—HRP Labeled Polymer Anti-Rabbit (K4003) (DAKO Corp.). For evaluation of toxicity, the thyroid and stomach were resected and frozen sections were stained with hematoxylin and eosin.

### Statistical Analysis

Comparisons of the values between 2 groups were performed using an unpaired *t* test. Statistical analyses were performed using SPSS (version 19.0), and probability values of less than 0.05 were considered to denote statistical significance. Survival analysis was performed using the Kaplan–Meier method, and the log-rank test with Holm correction was used for the group comparison.

## RESULTS

**FIGURE 1.**
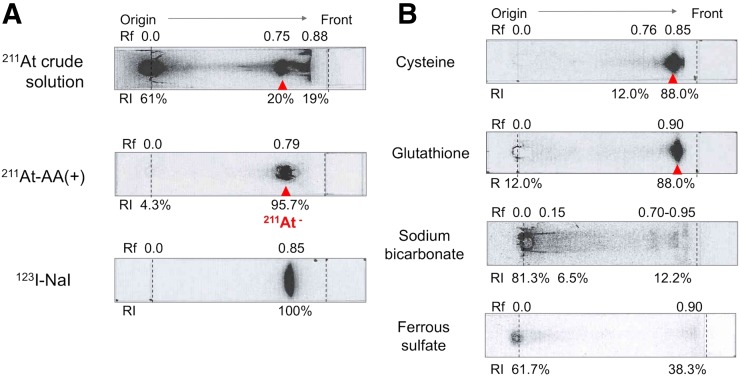
(A) TLC profiles of ^211^At solutions and ^123^I-NaI solution. (B) TLC profiles of ^211^At solutions after addition of various reducing agents (1 w/v%).

TLC analysis depicted that the crude ^211^At solution was a mixture of at least 3 chemical species of ^211^At, as shown in Figure 1A. Although AA(+) was composed of a single chemical species of ^211^At (Rf, 0.79) with a high radiochemical yield (95.7%), this species showed a similar TLC profile to that of ^123^I-NaI (I^−^; Rf, 0.85). Figure 1B shows the TLC profiles of the ^211^At solutions after addition of various reducing agents. Cysteine and glutathione also provided ^211^At-asatide ions of high radiochemical purity, but the purity was less than that under the AA(+) condition. On the other hand, sodium sulfite and ferrous sulfate had no significant effect on the TLC profiles of the crude ^211^At solutions under these conditions. Among the various reducing agents, addition of AA provided a ^211^At solution of the highest radiochemical purity, and it was stable for 24 h. We used AA as the reducing agent for further experiments.

**FIGURE 2.**
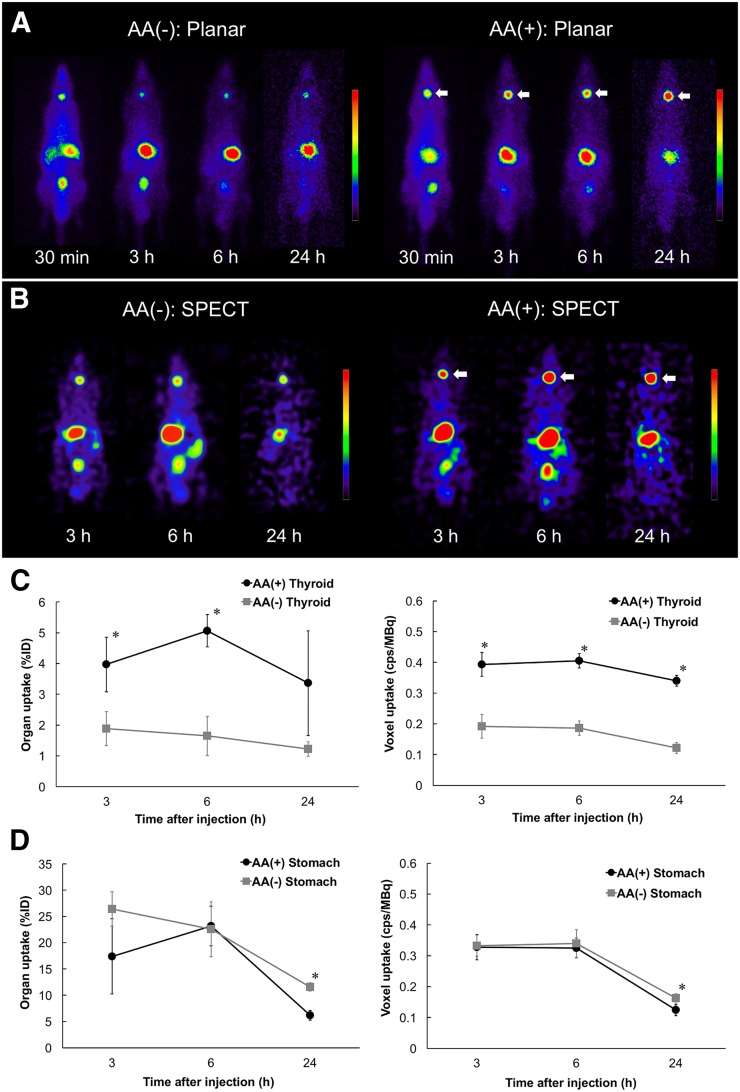
(A and B) Planar (A) and SPECT (B) images of normal rats: AA(−) group and AA(+) group. Increased uptake was observed in thyroid gland (arrows) under AA(+) condition as compared with AA(−) condition. (C and D) Time–activity curves in thyroid (C) and stomach (D) after injection of ^211^At solution on planar and SPECT (cps/MBq) images. %ID = percentage injected dose. *P < 0.05.

Whole-body distributions of ^211^At in the normal rats are shown in Figure 2. High uptake was observed in the thyroid, stomach, and bladder in both planar and SPECT images. Uptake by the thyroid was significantly better after administration of AA(+) than after administration of AA(−). Measurement using the γ-counter also showed high accumulation and significantly enhanced thyroid uptake after injection of AA(+) as compared with AA(−) (Table 1).

**TABLE 1:** Comparison of Whole-Body Distribution After Intravenous Administration of ^211^At Solutions Between AA(−) Group and AA(+) Group

Site	AA(−)	AA(+)	*P*
Brain	0.03 ± 0.02	0.02 ± 0.00	0.89
Thyroid	61.7 ± 16.2	417.6 ± 108.3	0.03*
Submandibular gland	0.20 ± 0.01	0.17 ± 0.02	0.44
Heart	0.24 ± 0.02	0.24 ± 0.03	0.25
Lung	0.78 ± 0.18	0.84 ± 0.05	0.28
Thymus	0.20 ± 0.02	0.35 ± 0.06	0.02*
Liver	0.21 ± 0.07	0.11 ± 0.00	0.75
Stomach	3.36 ± 1.16	3.56 ± 0.15	0.18
Small intestine	0.31 ± 0.06	0.30 ± 0.02	0.25
Large intestine	0.29 ± 0.01	0.56 ± 0.13	0.15
Cecum	0.36 ± 0.05	0.41 ± 0.08	0.14
Kidney	0.33 ± 0.03	0.29 ± 0.02	0.40
Adrenal gland	0.19 ± 0.04	0.13 ± 0.01	0.45
Pancreas	0.15 ± 0.01	0.13 ± 0.05	0.55
Spleen	1.13 ± 0.22	1.36 ± 0.28	0.14
Testis	0.35 ± 0.01	0.35 ± 0.02	0.25
Blood	0.12 ± 0.02	0.13 ± 0.00	0.24

* Statistically significant.

Data are mean (±SE) percentage injected dose per gram.

**FIGURE 3.**
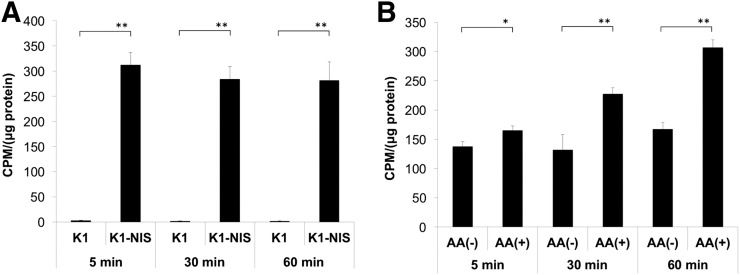
(A) In vitro uptake analysis of ^211^At uptake in K1 and K1-NIS cells. (B) Comparison of ^211^At uptake in K1-NIS cells between AA(−) and AA(+). CPM = count per minute. *P < 0.05. **P < 0.01.

Cellular uptake analysis showed high uptake of ^211^At in the K1-NIS cells but almost no uptake in the K1 cells (Fig. 3A), suggesting that ^211^At is transported into differentiated thyroid cancer cells through NIS. Uptake in the K1-NIS cells was significantly increased under the AA(+) condition as compared with the AA(−) condition (Fig. 3B).

**FIGURE 4.**
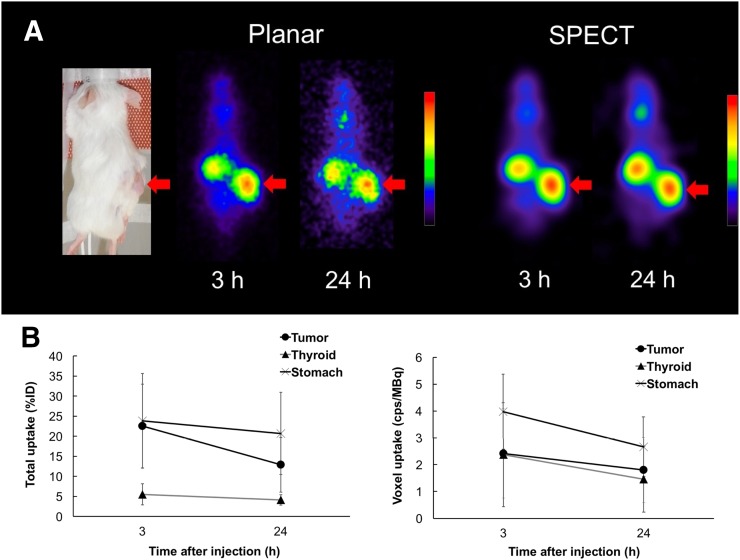
(A) Planar and SPECT images of mouse K1-NIS xenograft model after injection of AA(+). High uptake was observed in xenografts (arrows). (B) Uptake of ^211^At in tumor xenograft, thyroid, and stomach at 3 and 24 h after injection of AA(+). %ID = percentage injected dose.

The mouse tumor xenograft model revealed high accumulation in the K1-NIS tumor at 3 and 24 h after administration (Fig. 4A). Planar images revealed that the activity concentration of ^211^At was 22.5 ± 10.4 percentage injected dose at 3 h and 12.9 ± 6.8 percentage injected dose at 24 h in the K1-NIS xenograft. The equivalent dose in the tumor was estimated to be 9.7 ± 7.0 Gy. Measurement of the radioactivity in the K1-NIS xenografts (*n* = 3) at 24 h after the injection of ^211^At is shown in Table 2.

**TABLE 2:** Whole-Body Distribution After Intravenous Administration of AA(+) in Mouse K1-NIS Xenograft Model (*n* = 3)

Site	Data
Thyroid	101.9 ± 27.0
Salivary gland	10.4 ± 6.1
Liver	2.8 ± 0.8
Stomach	22.1 ± 10.9
Kidney	3.6 ± 0.7
Pancreas	2.6 ± 0.4
Spleen	4.6 ± 1.6
Testis	2.7 ± 0.1
Lung	10.6 ± 3.5
Small intestine	2.5 ± 0.3
Tumor	8.3 ± 3.1

Data are mean (±SE) percentage injected dose per gram.

**FIGURE 5.**
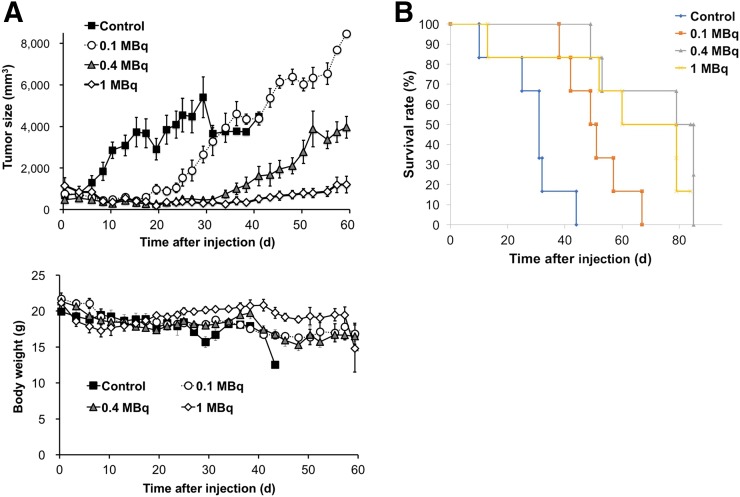
Treatment response after administration of AA(+). (A) Change in tumor size and body weight after administration of AA(+). (B) Kaplan–Meier analysis for comparison of survivals.

The change in tumor size after administration of ^211^At is shown in Figure 5A. Tumor growth was inhibited immediately after administration of ^211^At, and the treatment effect was dose-dependent. In the 1-MBq group, tumor growth was suppressed until approximately 40 d after the injection and regrowth was relatively slow. With regard to body weight, there was a slight drop in the 1-MBq group as compared with the other groups (Fig. 5B). However, this effect was transient, with the body weight restored within 2 wk and remaining relatively stable thereafter. For the survival analysis, the ^211^At administration groups (0.1, 0.4, and 1 MBq) showed significantly better survival rates than the control group (Fig. 5C).

**FIGURE 6.**
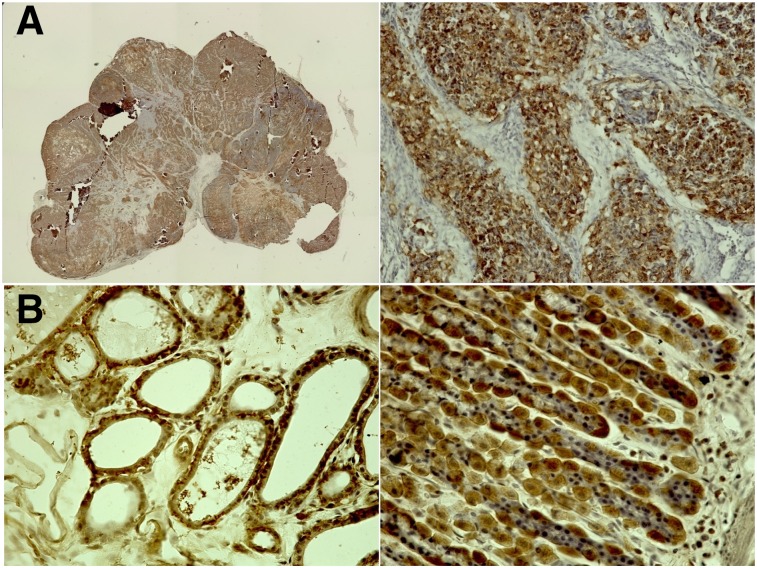
Immunohistochemical staining for NIS (anti-SLC5A5 rabbit IgG): K1-NIS xenograft at low (left) and high (right) magnifications (A); thyroid (left) and stomach wall (right) of normal rat (B). Cells showing NIS expression are stained brown.

Expression of NIS was confirmed in the K1-NIS tumor xenografts, thyroid, and stomach wall by immunohistochemical staining (Fig. 6).

## DISCUSSION

In the present study, we demonstrated that the radiochemical purity of ^211^At was increased by treatment with AA. Uptake of AA-treated ^211^At was enhanced in the NIS-expressing cells in in vitro studies and in the normal thyroid after intravenous administration.

AA(+) showed a dose-dependent treatment effect in the K1-NIS xenograft models. ^211^At is a very promising α-emitter as it can be produced in an accelerator and has similar chemical properties to iodine (10). However, one of the major problems of astatine is that there is no stable isotope and the basic chemistry is poorly understood (18). In previous publications on ^211^At, the oxidation states of aqueous solutions of astatine, such as At^−^, At(0), HOAt, AtO_3_^−^, and H_5_AtO_6_, vary (19). In the present study, we found that the oxidation states of ^211^At converged into a single component, which is assumed to be astatide ion (At^−^) according to the TLC analysis using ^123^I and ^211^At. ^211^At-astatide ions are labile in aqueous solutions and easily oxidized to higher oxidation states (18–20). Several reducing agents, including AA, were useful for the preparation of ^211^At-astatide ions in aqueous solution and for stabilization. TLC analyses of the solutions are desired, since the quality of the solutions vary.

In a previous study in which the distribution of astatine was evaluated, high uptake was observed in the thyroid, stomach, lungs, and spleen (10,18). Astatine is actively transported across the plasma membrane into the cytoplasm via NIS (12). The thyroid and stomach also showed a high accumulation of iodine, reflecting expression of NIS (18). These results were compatible with the whole-body distribution in the normal rats of our study, suggesting that NIS is the key symporter in the uptake process of astatine.

As shown in Figure 6, stomach cells also showed NIS expression. However, no significant increase of ^211^At uptake was observed in the stomach after administration of AA(+) as compared with AA(−). Spetz et al. reported the difference in the whole-body distribution between ^211^At and ^125^I/^131^I (18). In their study, decreased uptake of ^211^At by the thyroid and increased uptake in the stomach were observed as compared with ^125^I/^131^I. Their study suggested that the transport mechanism of ^211^At via NIS might differ between the thyroid and the stomach. There are some differences in NIS expression by glycosylation modification, dimerization, and transcriptional regulation (including epigenetic regulation) (21). It is possible that these factors or some other unknown mechanisms might affect the transport function of NIS for astatide, explaining the difference in uptake between the stomach and the thyroid.

For K1-NIS tumor, we performed a cellular uptake analysis to compare ^211^At uptake between AA(+) and AA(−), in which uptake was normalized by the number of cells. Uptake of ^211^At was significantly increased under the AA(+) condition as compared with the AA(−) condition in K1-NIS cells (Fig. 3B), as well as in the thyroid glands of normal rats (Fig. 2). Meanwhile, we need to consider the ethical 3R rules (replacement, reduction, and refinement) in animal experiments. Therefore, we thought it would be precise and ethically reasonable to compare by simultaneous cellular uptake analysis.

Petrich et al. evaluated the treatment effect of ^211^At in K1-NIS xenograft mice (12). In their study, a total activity of 2.5 MBq was intraperitoneally administered in 3 fractions within 16 d. Although they stated that ^211^At^−^ was used in their experiments, there was no description of any detailed analysis of the ^211^At solutions used in their study. In our study, we evaluated the oxidation states of ^211^At in solution in greater detail and proved that appropriate preparation of the astatine solution using reducing agents, such as AA, is important for maximizing the treatment effect of ^211^At in the form of astatide. They also showed that fractionated ^211^At therapy resulted in complete remission of the K1-NIS xenografts and also stated that a single administration caused stable shrinkage for up to 8 wk but was followed by tumor regrowth. We observed tumor shrinkage for up to 41 d after the administration of ^211^At (1 MBq), followed thereafter by regrowth, similar to the findings of the aforementioned study. They also reported that administration of a total of 2.5 MBq of ^211^At caused moderate damage in the thyroid by showing decreased uptake in ^99m^TcO_4_ scintigraphy and atrophy in the histologic analysis. We showed that a smaller dose (0.1 MBq) of ^211^At is also effective for suppression of tumor growth, although the regrowth was faster than after administration of the higher dose (0.4 MBq or 1 MBq). These results suggest that a more fractionated administration can be performed during targeted α-therapy using ^211^At in the clinical setting to reduce the side effects in normal organs. Fractionated administration of an α-emitter has been successfully adapted in ^223^Ra therapy for bone metastasis in castration-resistant prostate cancer (22). Therefore, targeted α-therapy with ^211^At would be clinically feasible, and the short half-life of ^211^At (7.2 h) is more ideal for preventing potential side effects after repeated administration over a short period.

Besides differentiated thyroid cancer, there are other cancers that show expression of NIS, such as breast cancer and gastric cancer (23,24). In patients with these cancers, ^211^At therapy can be one of the treatment options, especially for those with multiple metastases, in combination with appropriate iodine blocking of the normal thyroid (25). Recently, other groups in Japan have successfully demonstrated the excellent treatment effect of ^211^At-labeled antibody or compounds for peritoneal dissemination from gastric cancer and pheochromocytoma (8,9). Appropriate targeting is our next challenge, with careful evaluation of dehalogenation of the ^211^At compounds (13).

In the present study, the structure of the follicle was disrupted in the thyroid tissue at 3, 7, and 15 d after the administration of AA(+), whereas no significant difference was observed in the structure of gastric mucosa (Supplemental Fig. 1; supplemental materials are available at http://jnm.snmjournals.org). It was suggested that ^211^At could ablate the thyroid tissues, but no significant effect was observed in the stomach. There are some publications about the chronic toxicity of ^211^At. Cobb et al. reported that spleen, lymph nodes, bone marrow, gonads, thyroid glands, salivary glands, and stomach were affected after administration of ^211^At in mice (61 kBq/g of body weight) (26). However, most of them showed a transient change at 14 d and returned to normal levels or resulted in minimal damage at 56 d after administration, except for thyroid glands and gonads. The normal thyroid glands in thyroid cancer patients are usually removed or ablated in the clinical setting and thus would not be a problem. For the gonads, treatment depends on the tolerability of the patients. Petrich et al. reported that thyroid atrophy was observed in all mice and that inflammation of the lungs and stomach or bowel were observed in some mice during 1 y of follow-up after a total administration of 2.5 MBq of ^211^At (12). It was suggested that no critical finding was observed with the administered dose below 1 MBq.

This study had several limitations. First, we did not evaluate the side effects of ^211^At, other than the thyroid and stomach. Detailed analysis of the side effects, especially in relation to the dose dependency, is the next important subject for study before clinical application. Second, we did not perform fractionated administration of ^211^At in this study. This is also another important prerequisite to clinical application, as well as for evaluation of the side effects. Third, we preliminarily performed the in vitro cellular survival assay using K1-NIS cells and confirmed dose-dependent decreased cell viability in AA(+) compared with ^131^I (Supplemental Fig. 2). Comparison of the effectiveness of ^211^At and ^131^I is essential to prove that ^211^At therapy is more beneficial for patients who are refractory to repetitive ^131^I treatment with preserved uptake ability of NIS.

## CONCLUSION

This study revealed that an increase in the radiochemical purity of astatide in ^211^At solution by addition of AA was associated with significantly enhanced uptake of ^211^At by both normal thyroid tissue and differentiated thyroid cancer cells. The treatment effect of ^211^At solution in the K1-NIS xenograft model was dose-dependent and associated with prolonged survival, suggesting the potential applicability of targeted α-therapy for the treatment of advanced differentiated thyroid cancer.

## DISCLOSURE

This study was funded by KAKENHI (C) (15K09955) from the Ministry of Education, Culture, Sports, Science, and Technology (MEXT) and by the QiSS program of the OPERA from the Japan Science and Technology Agency (JST). No other potential conflict of interest relevant to this article was reported.

## KEY POINTS

**QUESTION:** Is targeted α-therapy using astatine (^211^At) useful for the treatment of differentiated thyroid cancer?
**PERTINENT FINDINGS:** Astatide solution with ascorbic acid showed dose-dependent treatment effect with minimal toxicity.
  It showed lower cell viability than ^131^I, suggesting new effective treatment in thyroid cancer.
**IMPLICATIONS FOR PATIENT CARE**: ^211^At can be a useful treatment option in differentiated thyroid cancer.
