# Supplementary material for: Enhancement of 211At Uptake via the Sodium Iodide Symporter by the Addition of Ascorbic Acid in Targeted α-Therapy of Thyroid Cancer
Source: J Nucl Med. 2019 Sep;60(9):1301–7. doi: 10.2967/jnumed.118.222638 (PMC6735285; doi:10.2967/jnumed.118.222638)
Supplement: Supplementary file 1 [file jnm222638SupplementalData.pdf]

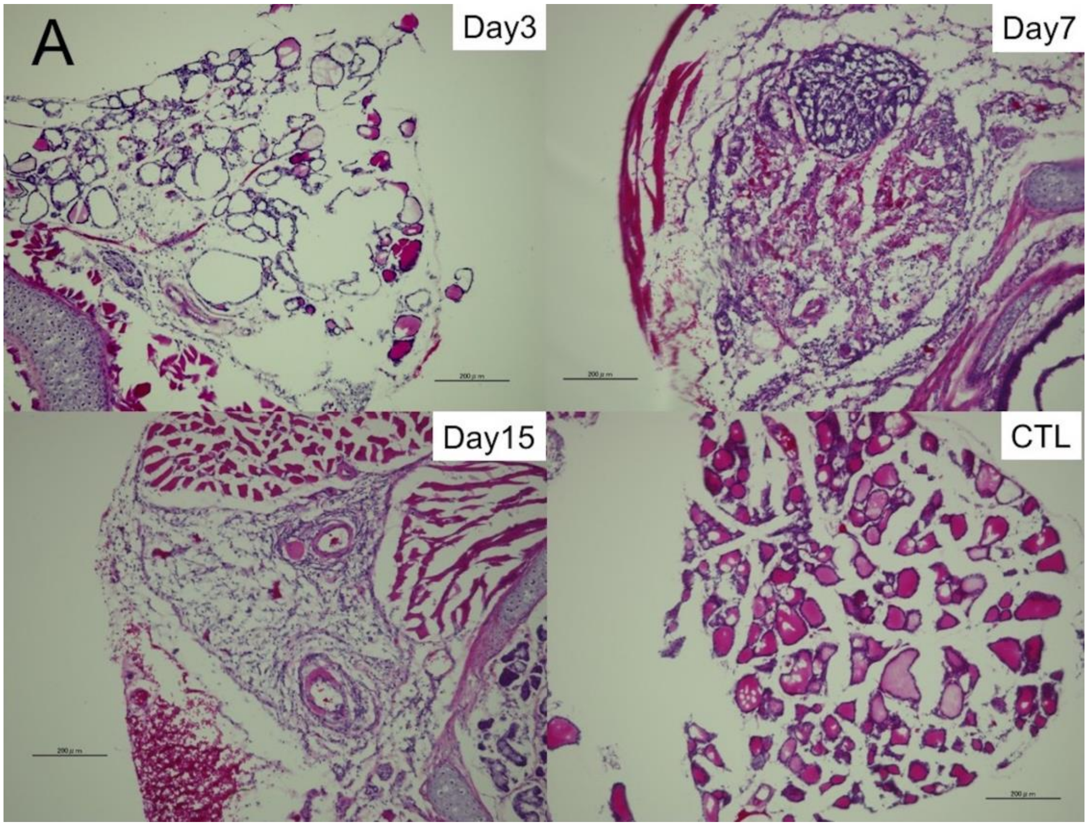
A
Day3
Day7
Day15
CTL
200 μm
200 μm
200 μm
200 μm

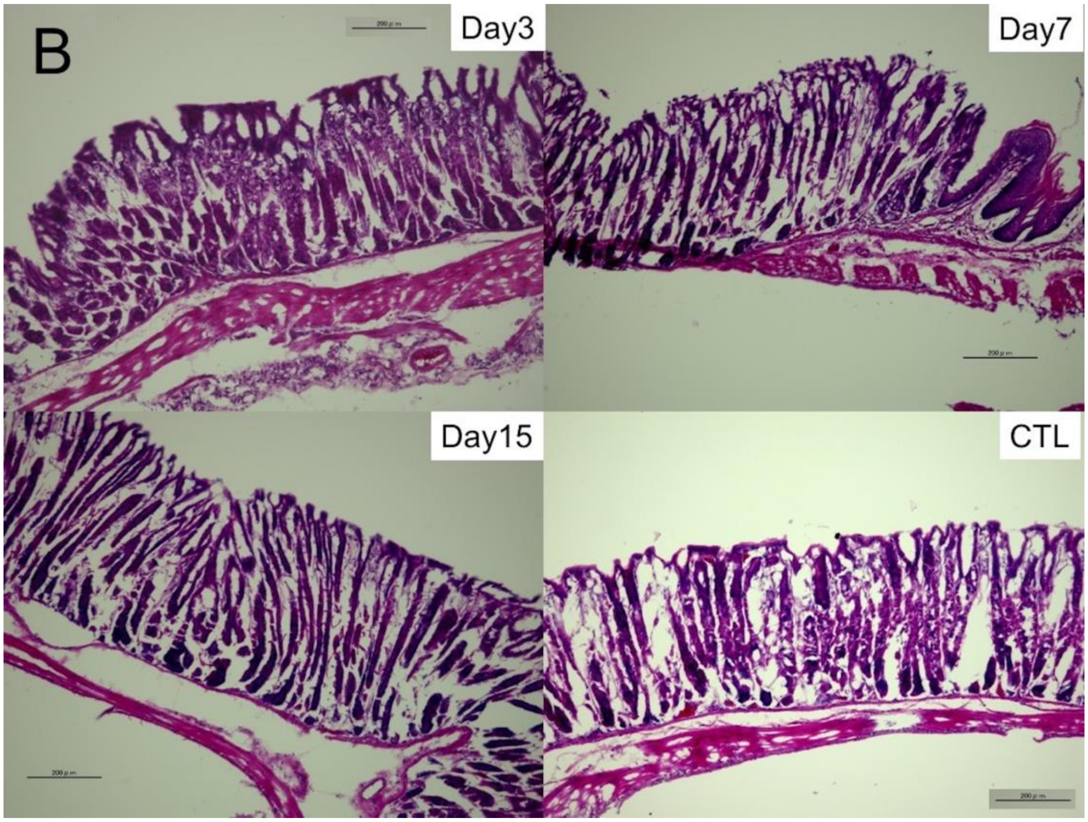

Supplemental figure 1. H&E staining of the (A) thyroid gland and (B) stomach in normal ICR mice at 3, 7 and 15 days after administration of AA(+) ^{211}At solution (1 MBq) and control (CTL). Bar indicates 200µm.

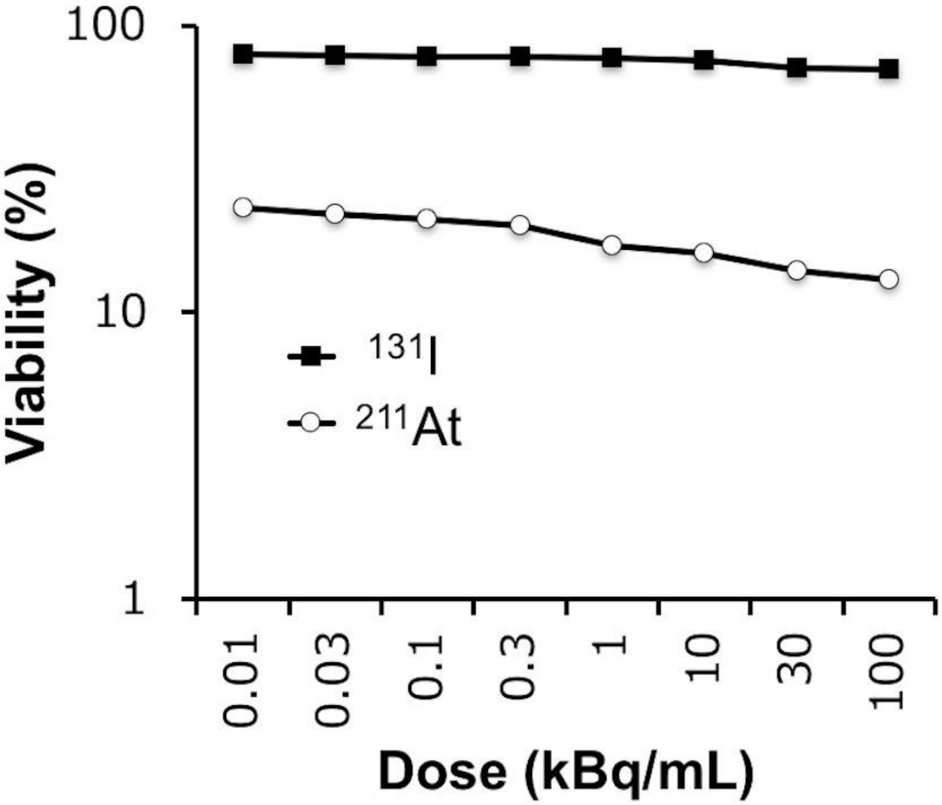

Supplemental figure 2. *In-vitro* cellular survival assay using K1-NIS cells for the comparison between ^{131}I and AA(+) ^{211}At.
